# Cost of gastroenteritis in Australia: A healthcare perspective

**DOI:** 10.1371/journal.pone.0195759

**Published:** 2018-04-12

**Authors:** S. Fiona Barker, Ella Zomer, Joanne O’Toole, Martha Sinclair, Katherine Gibney, Danny Liew, Karin Leder

**Affiliations:** 1 Department of Epidemiology and Preventive Medicine, School of Public Health and Preventive Medicine, Monash University, Melbourne, Victoria, Australia; 2 The Peter Doherty Institute for Infection and Immunity, The University of Melbourne, Melbourne, Victoria, Australia; Australian National University, AUSTRALIA

## Abstract

**Background:**

Acute gastroenteritis illness is a common illness that causes considerable morbidity, but current estimates of the cost to the Australian healthcare system are unknown.

**Objective:**

To estimate the current healthcare utilisation and direct public healthcare system costs attributable to acute gastroenteritis illness in Australia.

**Methods:**

This is an incidence-based cost-of-illness study focused on quantifying direct health care costs using a bottom-up approach. Data on general practitioner consultations, prescribed medications, diagnostic tests, specialist consultations, emergency department visits and hospital admissions were collected from national reports.

**Results:**

Using 2016 prices, the estimated annual direct per capita cost of acute gastroenteritis illness was AUD$14.87 (USD$10.71), equating to AUD$20.27 (USD$14.59) per case. The estimated overall economic burden in Australia was AUD$359 million (USD$258 million; AUD$1.5 million per 100,000 people). The major contributors to this cost were hospital admissions (57.1%), emergency department visits (17.7%), and general practitioner consultations (14.0%). Children under five years of age have the highest per capita rates of acute gastroenteritis illness; however, service utilisation rates vary by age group and both young children and older adults accounted for a substantial proportion of the overall economic burden attributable to acute gastroenteritis illness.

**Conclusions:**

Although chronic diseases comprise a large cost burden on the healthcare system, acute illnesses, including acute gastroenteritis illness, also impose substantial direct healthcare system costs. Providing data on current cost estimates is useful for prioritizing public health interventions, with our findings suggesting that it would be ideal if targeted interventions to reduce hospitalisation rates among young children and older adults were available.

## Introduction

Acute gastroenteritis illness (AGI) is a major cause of morbidity and mortality worldwide. In the past decade, studies have found community incidences of AGI ranging from 0.274 episodes per person per year (pppy) in 2008/9 in the UK [1] to as high as 1.4 in both Denmark [2] and USA [3], although some of this variation is likely due to differences in the definitions used [4, 5]. Healthcare seeking behaviour also varies across countries and studies, with estimates ranging from 6.5% [1] to 39.3% [6] of AGI cases seeking treatment from a general practitioner (GP), and hospitalisation rates between 0.3% [7] and 4.6% [8].

Using the latest information on AGI incidence in Australia (0.74, 95% CI 0.64–0.84 episodes pppy; [9, 10]), Gibney et al. [11] estimated a total of 16.6 million cases in 2010. Earlier work estimated AGI incidence as 0.8 episodes pppy in 1999 [12], 0.92 in 2002 [13] and 0.77 in 2007/8 [14]. The change in incidence is likely due to a range of factors including differences in methodology. However, it may also reflect a true decrease in incidence of pathogens causing gastroenteritis, such as rotavirus [10], following the roll-out of the rotavirus vaccine in 2007 [15, 16].

The direct costs associated with AGI also vary across countries and studies, including estimates of AUD$5 per case in 1999 in Australia [12], €14 per case in 1999 in the Netherlands [17], CAN$47 per case in 2004 in Canada [18, 19], and €56 per case in 2013–14 in Sweden [20]. In addition to methodological differences, the variation in cost estimates likely reflects a wide range of differences including service utilisation, care pathways, costs, and healthcare system structures.

Across the Australian cost of illness (COI) studies, different estimates of healthcare service utilisation have been reported. For example, reports suggest that 12.9% of AGI cases visited a doctor in 1999 [12], 27.8% in 2007/8 [14], and 15.2% in 2008/9 [21]; 0.15% of cases visited a specialist in 1999; 0.11% of cases were admitted to hospital in 1999 and 3.7% in 2007/8. In 76.5% of GP consultations in 1999, patients presenting with AGI were provided with some form of medication, and in a 2008/9 estimate, 37% of all AGI cases used some form of medication. Reports also suggest that between 1.7% (1999) and 3.2% (2008/9) had pathology tests. Most of these differences are likely to reflect changes in practice as they were based on surveys using similar methodologies [12, 21], but the 2007/8 study may not be nationally representative as it was a survey of South Australia only.

To the best of our knowledge, current estimates of the costs associated with AGI in Australia are lacking, with previous estimates employing data from nearly 20 years ago [12]. Therefore, the objectives of this study were to quantify the current healthcare resource use of patients with AGI, and the associated costs, from the perspective of the Australian public healthcare system. This approach was adopted as a conservative estimate to inform health policy makers whose main interest lies in the financial burden to the healthcare system.

## Methods

An epidemiological model was developed to estimate the total cost of AGI in Australia, from a healthcare system perspective. A bottom-up approach was employed using nationally-representative incidence data to estimate the total number of cases of AGI in Australia and applying the associated economic cost per case. Economic costs were based on estimations of direct healthcare resource use and included general practitioner (GP) consultations, diagnostic tests, prescribed medications, specialist consultations, emergency department (ED) visits and hospitalisations. Costs borne by individuals, such as gap payments (additional costs charged by GPs or for medications), private healthcare costs, and other out-of-pocket expenses, were not included. Further, indirect costs such as transport to or from hospital and costs attributed to productivity loss were also excluded. All costs were reported in 2016 Australian dollars and for the major findings, these values were converted to United States dollars assuming a conversion of 1 to 0.72 [22], as per previous studies [23–25].

### Data sources

Multiple secondary data sources were used to estimate the cost of AGI in Australia.

The incidence of AGI in Australia was determined using data from the National Gastroenteritis Survey II (NGSII), a computer-assisted telephone survey of 7,578 individuals across all states and territories of Australia during 2008–2009. Respondents were asked questions about diarrhoea or vomiting in the previous four weeks, with infectious gastroenteritis defined as experiencing at least three loose stools or two vomits in 24 hours, or, if the person had concomitant respiratory symptoms, gastroenteritis was defined as at least four loose stools and/or at least three vomits in 24 hours [10].

Primary healthcare service use was determined from The University of Sydney’s ‘Bettering the Evaluation And Care of Health’ (BEACH) program, a cross-sectional national study that randomly surveyed approximately 1,000 GPs each year and recorded details on 100 consecutive consultations including type of consultation, prescribed medications, diagnostic tests, and referrals to specialists. The resultant annual database of approximately 100,000 records is considered generalisable of visits to GPs nationally. Our analysis was based on a data extraction of GP consultations with at least one AGI problem, based on ICPC-2 (international classification for primary care) rubric classifications D70 and D73 (1.29% of all GP encounters surveyed) from April 2011 to March 2016 [26]. New cases, defined as “the first presentation of a problem, including the first presentation of a recurrence of a previously resolved problem but excluding the presentation of a problem first assessed by another provider” [26], and follow-up visits were included. The number of non-admitted AGI-related ED visits was estimated from a range of data sources including the Australian Institute of Health and Welfare (AIHW) hospital statistics report for 2014–15 [27]. The number of AGI-related hospital admissions was estimated from age-stratified annual hospitalisation rates for AGI from the AIHW National Hospital Morbidity database using ICD-10 codes (A00-A09) [28].

Medicare is part of Australia’s universal health insurance scheme and costs for primary healthcare service use were obtained from the Medicare Benefits Schedule (MBS) [29] including GP consultations, specialist consultations and diagnostic tests. Prescription medication costs borne by the healthcare system were obtained from the Pharmaceutical Benefits Scheme (PBS), an Australian government initiative which subsidises medications to make them more affordable to patients [30]. Reimbursed costs were obtained as the Dispensed Price for Maximum Quantity (DPMQ), specific to medication strength and formulation.

The cost of ED visits was determined from the National Hospital Cost Data Collection 2014–2015 –Appendix 16 in [31], the annual collection of public hospital data, using gastrointestinal system illness urgency-related groups (URGs) for non-admitted separations. Similarly, the cost of hospital admissions was determined using Australian Refined Diagnosis Related Groups (AR-DRG), where each AR-DRG represents a class of patients with similar clinical conditions requiring similar hospital services. Average hospital costs for AR-DRGs are estimated annually from a representative sample of public hospitals, and include costs of imaging, pathology, resource use in the intensive care unit (ICU) and operating theatre, and prescribed pharmaceuticals. For our study, costing data were extracted from the National Hospital Cost Data Collection 2014–2015 –Appendix 3 in [31]. Costs for both ED visits and hospital admissions were inflated to 2016 prices using the health price index (HPI) [32].

Institutional ethics approval was not required for this study as only existing de-identified data collections were used for modelling.

### Sensitivity analysis

To account for parameter uncertainty, we performed both deterministic (one-way) and probabilistic sensitivity analyses. In the one-way sensitivity analysis, item numbers (i.e. number of GP visits, number of medications, etc.) and costs were varied (±25% of base case) to assess which had the most influence on the total cost. The probabilistic sensitivity analysis (PSA), based on 10,000 simulations, was performed in R (Version 3.2.0; RStudio Version 0.99.435) to assess the uncertainty in the key input parameters by varying them concurrently. Variables were represented by uniform distributions using ±25% as the minimum and maximum distribution values (refer to S3 Table). PSA results are reported as mean and 95th percentile values. For number of GP visits, 95% confidence intervals were available; these values were also used in the sensitivity analyses.

## Results

### Incidence of AGI

Using age-, sex- and state-weighted incidence of AGI from NGSII-2008 [9], together with population data for June 2016 [33], we estimated a total of 17,704,439 cases of AGI in Australia in 2016 (Table 1). The annual per capita rate of AGI is highest in children under the age of 5 years (1.58 episodes pppy), and also high for people 20 to 34 years of age (1.00–1.30 episodes pppy). Adults of working age (between 20 and 64 years old) account for 63.9% of all AGI cases.

**Table 1:** AGI cases in Australia, 2016.

Age group (years)	Per capita rate of AGI–mean (cases pppy) [9, 10]	Population– 2016 [33]	AGI cases—2016	% of cases
0–4	1.58	1,566,363	2,469,701	13.9
5−9	0.82	1,530,238	1,262,444	7.1
10−14	1.01	1,442,711	1,458,650	8.2
15−19	0.42	1,484,355	627,132	3.5
20−24	1.30	1,678,190	2,175,938	12.3
25−29	1.07	1,780,200	1,913,585	10.8
30−34	1.00	1,784,731	1,789,411	10.1
35−39	0.63	1,611,731	1,013,670	5.7
40−44	0.77	1,620,955	1,255,842	7.1
45−49	0.82	1,609,109	1,313,346	7.4
50−54	0.52	1,543,039	796,138	4.5
55−59	0.41	1,479,062	603,442	3.4
60−64	0.35	1,313,597	458,489	2.6
65−69	0.21	1,184,544	253,875	1.4
70−74	0.20	898,925	182,123	1.0
75−79	0.07	654,690	47,993	0.3
80−84	0.18	460,079	82,661	0.5
85+	0.00	484,640	-	0.0
Total		24,127,159	17,704,439	100.0

### Costs—Primary healthcare service use

#### GP consultations

Using age-stratified data on the proportion of GP consultations for AGI cases, along with the total number of GP consultations for 2016 (146,493,011; [34]), we estimated 1,893,996 GP consultations for AGI in 2016 (S1 Table). Children less than 5 years old had the highest per capita rate of AGI-related GP consultations, but individuals aged 15 to 64 years accounted for 66.4% of all AGI-related GP consultations. Further, older adults (65+) had the highest rate of GP consultations, with an estimated 36.9% of those with AGI visiting a GP.

Individuals who did not access primary healthcare services were assumed to have no costs incurred to the public healthcare system. To estimate the average cost of an AGI-related GP consultation (Table 2), costs were weighted by the type (length) of consultation, as well as the registration status of the GP, where 82.5% of GPs were assumed to be vocationally registered [35]. Only GP consultations in GP settings were included in our analysis as these represented 95% of all direct GP encounters (i.e. excluded home visits and visits to residential aged care facilities). The weighted average cost ($36.89; Table 2) was adjusted to account for other problems (non-gastroenteritis related) managed at each GP consultation, an additional 39.2 problems for every 100 GP consultations [26], such that 71.8% of the cost was attributed to gastroenteritis. The final weighted average cost ($26.50) was applied to the estimated total number of GP consultations to determine the total cost of AGI-related GP encounters of $50,190,534.

**Table 2:** GP consultations for AGI, by type of consultation, and their weighted costs.

Type of GP consult’n	# of GP consult’ns^a^	% of GP consult’ns	Unit costs (AUD) by reg’n status^b^		Average cost per GP consult’n (AUD)^c^	Weighted cost per GP consult’n (AUD)	Matched Medicare item [29]^b^	
			R	N			R	N
Short	44	0.80	$16.95	$11.00	$15.91	$0.13	3	52
Standard	5003	90.85	$37.05	$21.00	$34.24	$31.11	23	53
Long	433	7.86	$71.70	$38.00	$65.80	$5.17	36	54
Prolonged	27	0.49	$105.55	$61.00	$97.75	$0.48	44	57
Total weighted average cost						$36.89		

^a^Of the 6,288 AGI-related GP consultations, 91.9% (n = 5,777) were direct encounters of which 5,507 (95%) were included in our determination of average cost.

^b^Registration status: R = vocationally registered, N = non-vocationally registered. Costs obtained from MBS [29].

^c^Average cost per GP consultation, where 82.5% of GPs were assumed to be vocationally registered.

#### Prescribed medications

Medications were only included when they were prescribed by a GP during an AGI-related GP consultation. Over-the-counter medication costs were assumed to be borne by the individual and therefore excluded from this analysis. Based on the BEACH dataset, 41.9 prescriptions were ordered per 100 GP consultations [26] resulting in an estimated 793,984 prescriptions for AGI in 2016.

To estimate the average cost of these medications, the top 20 brands of prescribed medications for AGI were identified from BEACH and the number of prescriptions for each dosage and formulation were extracted [26]. Generic drug costs were used, when available, as a conservative estimate of cost. For medications where strength and formulation were unknown, the weighted average cost of all known strengths and formulations was used. Where a medication was not listed on the PBS, the cost to the healthcare system was assumed to be zero (i.e. not government subsidised). The weighted average cost of prescribed medications was $16.59 and the total cost of prescribed medications for AGI in 2016 was estimated at $13,172,602 (see S2 Table for full costing information).

#### Diagnostic tests

AGI-related diagnostic tests order by a GP included both pathology and imaging tests, with imaging tests assumed to be investigative tests ordered to rule out alternate, often more severe, conditions. The BEACH dataset reported that in 18.1% and 0.45% of GP consultations, at least one pathology or imaging test, respectively, was ordered, while there was a total of 31.9 and 0.49 tests ordered per 100 GP consultations, respectively. Using the list of diagnostic tests ordered for AGI-related GP consultations in BEACH [26], costs of tests were determined by item numbers on the MBS [29] and, if more than one item number for a test was available, the average cost was used. The weighted average costs for AGI-related diagnostic tests were $40.80 for pathology tests (Table 3) and $128.89 for imaging tests (Table 4), resulting in total cost estimates of $24,692,155 and $1,203,461 for pathology and imaging tests related to AGI presentations to GPs in 2016, respectively.

**Table 3:** AGI-related pathology tests and their weighted costs.

Pathology tests [26]	# of tests [26]	% of tests	Item cost (AUD) [29]	Weighted cost per test (AUD)	Matched Medicare item [29]
Amylase	6	0.3%	$9.70	$0.03	66500
Anti nuclear antibodies	4	0.2%	$24.45	$0.05	71097
Blood test	4	0.2%	$9.70	$0.02	66500
C reactive protein	54	2.7%	$9.70	$0.26	66500
Calcium/phosphate/magnesium	4	0.2%	$13.65	$0.03	66506
Chemistry; other	19	0.9%	$9.70	$0.09	66500
Coagulation	3	0.1%	$52.45	$0.08	65129 & 65070
ESR	19	0.9%	$7.85	$0.07	65060
EUC	68	3.4%	$17.70	$0.60	66512
Faeces MC&S	991	49.3%	$52.90	$26.09	69345
Faeces test	41	2.0%	$32.90	$0.67	69345
Ferritin	20	1.0%	$18.00	$0.18	66593
Folic acid	4	0.2%	$23.60	$0.05	66840
Full blood count	172	8.6%	$16.95	$1.45	65070
Glucose tolerance	28	1.4%	$18.95	$0.26	66542
H pylori	158	7.9%	$77.65	$6.11	66900
Hepatitis serology	7	0.3%	$29.25	$0.10	69478
Hormone assay	8	0.4%	$30.50	$0.12	66695
Immunology; other	47	2.3%	$32.90	$0.77	71057
Lipids	17	0.8%	$11.65	$0.10	66503
Liver function	52	2.6%	$17.70	$0.46	66512
Microbiology; other	34	1.7%	$52.90	$0.90	69345
Multibiochemical analysis	78	3.9%	$17.70	$0.69	66512
Other pathology	31	1.5%	$9.70	$0.15	66500
Other test NEC (not elsewhere classified)	14	0.7%	$9.70	$0.07	66500
Simple test; other	14	0.7%	$9.70	$0.07	66500
Thyroid function	26	1.3%	$34.80	$0.45	66719
Urinalysis	5	0.2%	$20.55	$0.05	69333
Urine MC& S (Microscopy, Culture and Sensitivities)	65	3.2%	$20.55	$0.66	69333
Urine test	6	0.3%	$20.55	$0.06	69333
Vitamin B12	10	0.5%	$23.60	$0.12	66838
Total weighted average cost				$40.80	

**Table 4:** AGI-related imaging tests and their weighted costs.

Imaging tests [26]	# of tests	% of tests	Item cost (AUD)	Weighted cost per test (AUD)	Matched Medicare item [29]
Ultrasound; abdomen	15	48.4	$111.30	$53.85	55036
X-ray; abdomen	5	16.1	$47.60	$7.68	58903
CT scan; abdomen	5	16.1	$305.00	$49.19	56401 & 56407
Ultrasound; pelvis	2	6.5	$98.25	$6.34	55065
Ultrasound; abdomen upper	3	9.7	$111.30	$10.77	55036
X-ray; neck	1	3.2	$32.55	$1.05	average of 57945 & 57956
Total weighted average cost				$128.89	

### Costs—Secondary healthcare service use

#### Specialist consultations

The BEACH dataset reported that 0.75% of all AGI-related GP consultations had a referral to a specialist [26], of which we assumed 76.1% were within the public healthcare system in line with public/private hospital separations described below [36, 37]. This resulted in an estimated 10,775 referrals to specialists for AGI-related issues in 2016. Costs of referrals to specialists were taken from the MBS [29] (items 104 and 110) and the average cost ($118.23) was used to estimate the total cost of AGI-related specialist consultations of $1,273,853.

#### Emergency department visits

For this analysis, only non-admitted ED visits were costed, assuming that admitted cases would be captured under hospital admissions. ED data were available for both public [27, 38] and private [39–41] hospitals, and an estimate of the number of AGI-related ED visits was available for 2009/10 [9]. The total number of ED visits for 2015/16 was estimated from the per capita rate of 0.33 ED visits (including admitted and non-admitted cases) from 2013/14 and 2014/15 [27, 33, 39, 40] and the percentage of all ED visits attributable to non-admitted AGI cases was 1.62% [9, 41, 42]. A total of 120,255 AGI-related ED visits, without subsequent hospital admission, was estimated assuming that 93.2% of ED visits were to public hospitals [27, 40]. Relevant URGs were used to determine a weighted average cost per ED visit of $527.58, when inflated to 2016 costs (Table 5), and applied to determine the total cost estimate of AGI-related ED presentations of $63,443,924.

**Table 5:** ED visits for gastrointestinal system and digestive system illness urgency-related groups for non-admitted separations (URGs) and their weighted costs [31].

URGs	% of separations^a^ [43]	Item cost (AUD)	Weighted cost (AUD)
URG 97	4.3	$825.09	$35.14
URG 52	42.1	$624.32	$262.80
URG 62	50.3	$437.46	$219.96
URG 114	3.4	$287.43	$9.67
Total weighted average cost			$527.58

^a^Separations are defined as “cessation of an episode of care for a patient within the one hospital stay”.

#### Hospital admissions

A total of 78,997 hospital admissions (including public and private) for AGI were estimated in 2016 from age-stratified annual hospitalisation rates for AGI (2014–15) (ICD-10 codes A00-A09) [28], and population data [33]. Of these, 60,125 were estimated to be public hospital admissions (Table 6), assuming 76.1% of all hospital separations [36] were in public hospitals [37]. Children under 5 years of age and older adults (65+ years) had higher per capita rates of hospitalisation for AGI and accounted for 12.8% and 31.2% of all AGI hospital admissions, respectively. Further, amongst AGI cases, older adults (65+) had the highest rate of hospitalisations (3.31%). AR-DRGs were used to determine a weighted average cost per hospital admission of $3,407.55, inflated to 2016 costs (Table 7), and a total cost estimate of $204,878,654.

**Table 6:** AGI-related hospital admissions (public only).

Age group (years)	Average annual hospitalisation rate for AGI per 100,000 [28]	Hospital admissions for AGI (2016) # (%)^a^
0–4	643.2	7,668 (12.8)
5−9	178.2	2,075 (3.5)
10−14	106.9	1,174 (2.0)
15−19	186.0	2,102 (3.5)
20−24	281.0	3,589 (6.0)
25−29	269.9	3,656 (6.1)
30−34	259.9	3,530 (5.9)
35−39	226.5	2,778 (4.6)
40−44	225.7	2,784 (4.6)
45−49	220.6	2,702 (4.5)
50−54	244.9	2,876 (4.8)
55−59	275.1	3,096 (5.2)
60−64	336.4	3,364 (5.6)
65−69	414.6	3,738 (6.2)
70−74	536.1	3,668 (6.1)
75−79	709.7	3,536 (5.9)
80−84	957.8	3,354 (5.6.)
85+	1202.2	4,434 (7.4)
Total		60,125 (100.0)

^a^ Estimated using population data for 2016 –refer to Table 1 [33].

**Table 7:** AGI-related hospital separations by diagnosis-related group and their weighted costs.

Australian Refined Diagnosis Related Group (AR-DRGs) [31]	% of separations^a^	Item cost (AUD)	Weighted cost (AUD)
AR-DRG G67A: Oesophagitis and gastroenteritis with catastrophic or severe complication or comorbidity	31.4%	$6,261.79	$1,969.12
AR-DRG G67B: Oesophagitis and gastroenteritis without catastrophic or severe complication or comorbidity	68.6%	$2,098.26	$1,428.43
Total weighted average cost			$3,407.55

^a^Separations are defined as “cessation of an episode of care for a patient within the one hospital stay” [43].

### Total costs

The total public healthcare system cost of AGI for 2016 was estimated at AUD$359 million (USD$258 million) or AUD$1.5 million per 100,000 people (Table 8). Note that this cost includes public healthcare system costs only and does not include costs borne by individuals or other indirect costs such as productivity losses. The per capita cost was AUD$14.87 (USD$10.71) and the cost per case was AUD$20.27 (USD$14.59). The majority of the cost was incurred through hospital admissions (57.1%), ED visits (17.7%), and GP consultations (14.0%).

**Table 8:** Direct public healthcare costs of AGI in 2016.

Healthcare service	# (%) of cases^a^	Total cost (million AUD)	% of cost	Cost (AUD) per 100,000 population
# AGI cases	17,704,439			
# visiting a GP	1,893,996 (100%)	$50.191	14.0	$208,000
# prescription medications	793,984 (41.9%)	$13.173	3.7	$55,000
# pathology tests	605,127 (31.9%)	$24.692	6.9	$102,000
# imaging tests	9,337 (0.5%)	$1.203	0.3	$5,000
# referred to a specialist	10,775 (0.6%)	$1.274	0.4	$5,000
# visiting ED	120,255 (6.3%)	$63.444	17.7	$263,000
# admitted to hospital	60,125 (3.2%)	$204.879	57.1	$849,000
Total		$358.856	100.0	$1,487,000

^a^% of cases with GP consultations

### Age-specific costs

While children under 5 years have the highest per capita rate of AGI and AGI-related GP consultations, there have been substantial reductions in hospitalisations for AGI over time. A 35% decrease in hospitalisations for children under 5 was recorded in the first year after the rotavirus vaccine was added to the national childhood immunisation program schedule (2007–08), with a continuing slow decline or negligible increase through to 2014/15 [44]. This reduction aligns with previous estimates of rotavirus-attributable hospitalisations [16].

A substantial burden is also borne by older adults, with AGI cases among adults over 65 years of age having the highest rate of GP consultations and hospital admissions. While the per capita rate of AGI is low among those over 65 years, and only 3.2% of cases occur in this age bracket, more than 30% of hospitalisations are incurred by older adults.

**Fig 1:**
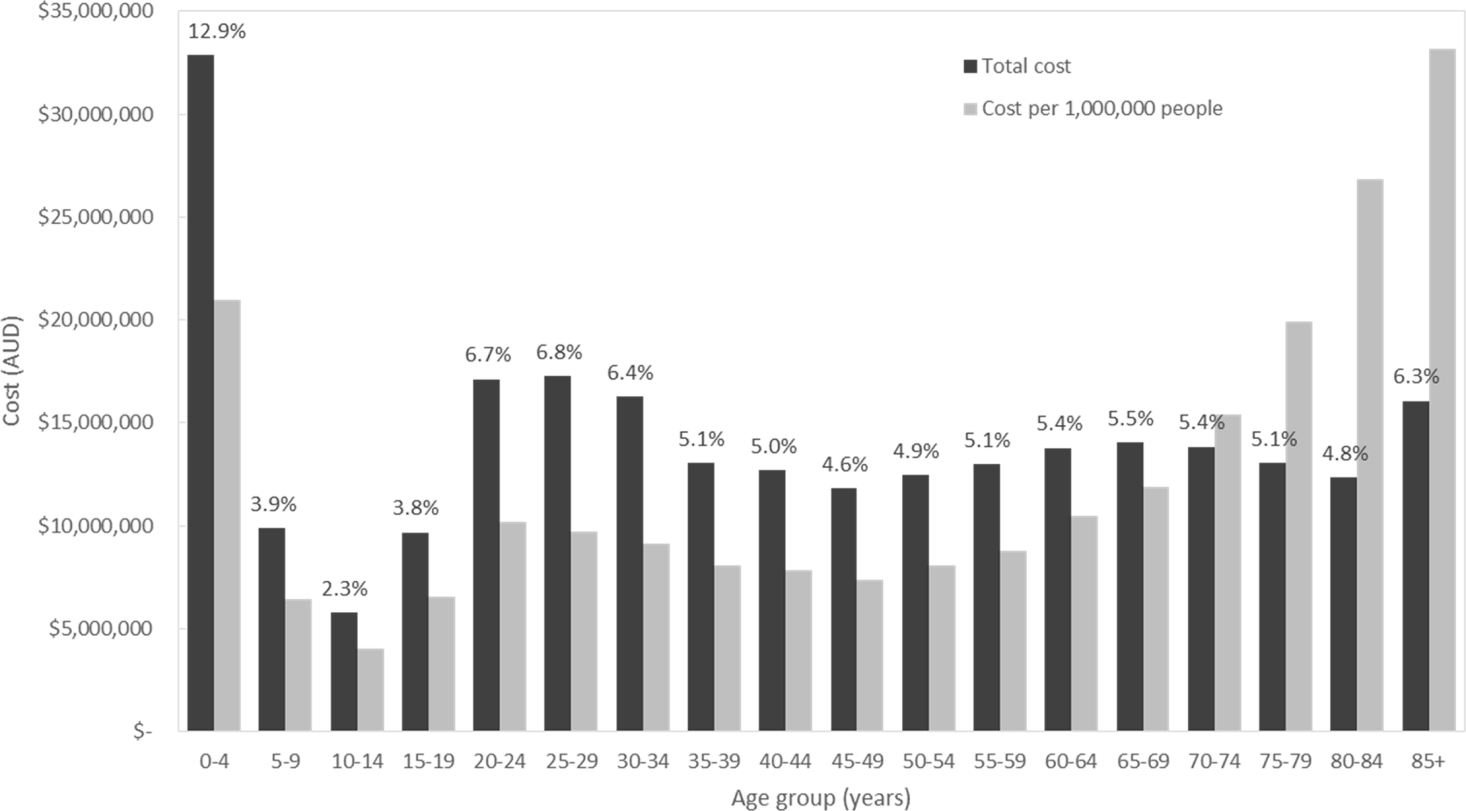
Combined costs (GP visits and hospitalisations) of gastroenteritis by age group. Black bars represent the combined costs by age group with percentage of total cost above the bar; grey bars represent population-weighted costs (cost per 1,000,000 people).

Hospital admissions and GP consultations account for nearly three quarters of the total cost of AGI. Assuming that service utilisation costs are the same for all ages, we estimated the combined cost of these two items by age group (Fig 1). As a percentage of the combined total cost of hospital admissions and GP consultations, the greatest cost was incurred by those aged 0–4 year (12.9%), 25–29 years (6.8%), 20–24 years (6.7%), 30–34 years (6.4%), and 85+ (6.3%). The per capita cost was greatest for individuals 85 years and older ($33.15), 80–84 years old ($26.86) and under 5 years of age ($20.98).

### Sensitivity analysis

**Fig 2:**
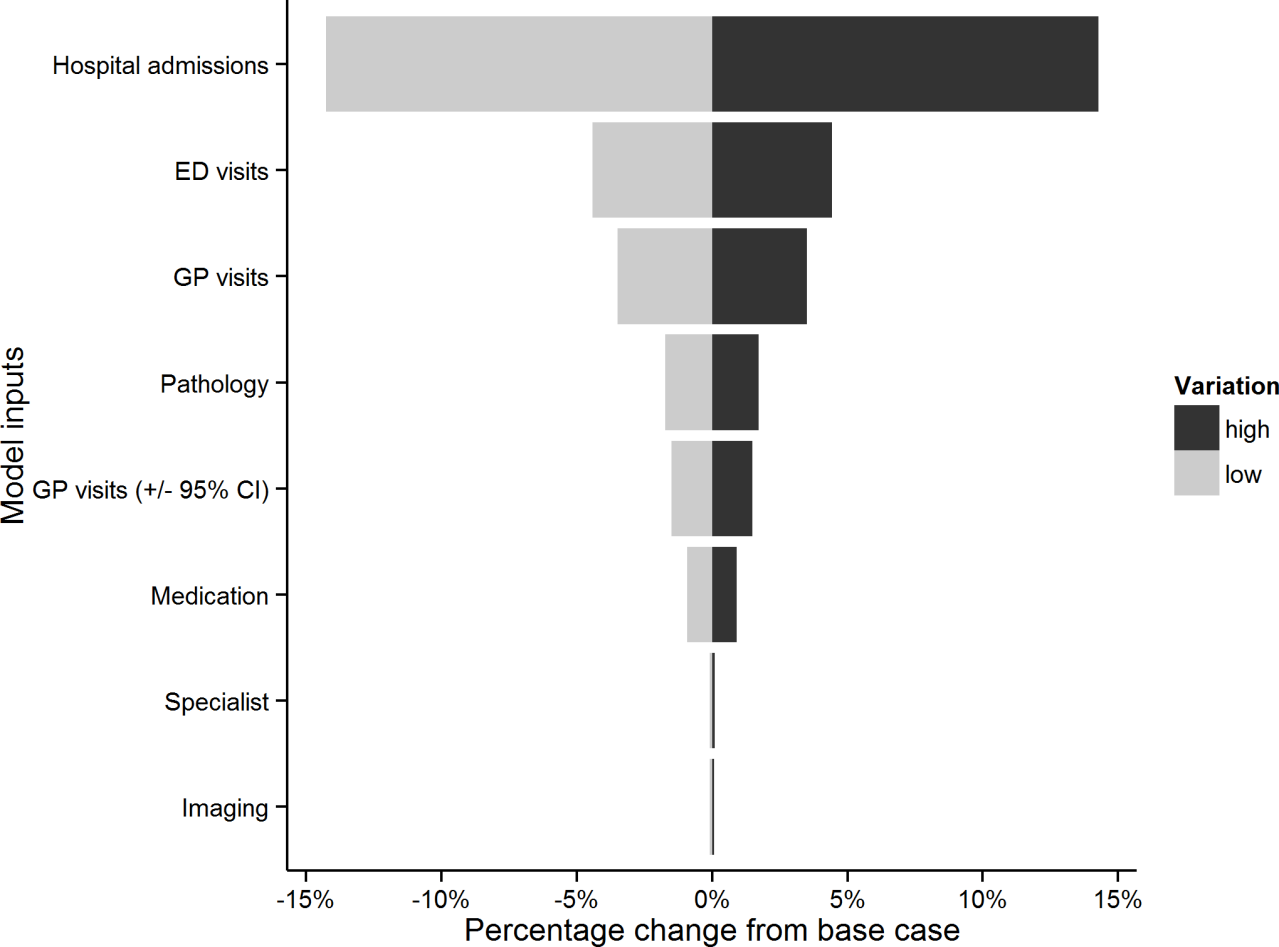
Tornado diagram summarizing the result of one-way sensitivity analysis with model inputs listed in descending order of variation of total cost.

In order to test the responsiveness of the model and the robustness of our results, we conducted a one-way sensitivity analysis. The variables in the sensitivity analysis were varied at a range of ±25% and the results are shown in a tornado diagram (Fig 2). The sensitivity analysis shows that hospital admissions and ED visits were the most influential parameters, followed by GP visits and pathology. Where 95% confidence intervals were used to describe the uncertainty around the number of GP visits (rather than ±25%), the variation in total cost was less than that from pathology tests.

The PSA resulted in an average total cost of $356 million ($279 million, $449 million).

## Discussion

This is the first study to estimate the direct cost of AGI in Australia in nearly 20 years. Direct healthcare system costs were estimated to be $359 million, or 0.3% of government health spending (based on 2014/15 spending) [45], with the largest costs incurred by hospital admissions, ED visits, and GP consultations.

Our findings show the cost per case of AGI in Australia in 2016 was AUD$20.27. In 1999, Hellard et al. estimated the direct costs of AGI at AUD$5.00 which, when inflated to 2016 prices, would be approximately AUD$7.73. The difference between these values reflects a combination of methodological changes and differences in service utilisation and care. While the per capita rate of AGI is now slightly lower (0.74 vs. 0.8), as is the rate of seeking treatment from a specialist, the rates of GP consultations, pathology tests, ED visits, and hospital admissions are higher (Table 9). Some of these differences (specialist visits and pathology tests) are related to changes in practice/behaviours, while others (GP consultations, hospital admissions, and ED visits) reflect changes in methodology for determination of the cost which may or may not include changes in practice.

**Table 9:** Use of healthcare services.

Healthcare services	% of AGI cases		% of AGI-related GP consultations	
	Australia, 1999 [12]	Australia, 2016	Australia, 1999 [12]	Australia, 2016
GP consultations	6.75	10.70		
Prescription medications	3.87	4.48	57.39	41.9
Over the counter medications	1.29	n/a	19.12	n/a
Pathology tests	0.90	3.42	13.35	31.9
Imaging tests	n/a	0.05	n/a	0.5
Specialist visits/Alt treatment	0.08	0.06	1.14	0.6
ED visits	0.17	0.68	2.54	6.3
Hospital admissions	0.10	0.34	1.51	3.2

We also found that there was a higher service utilisation burden of AGI incurred by young children and older adults, with 12.9% of combined costs from hospital admissions and GP visits incurred by children under 5 years of age and 27.2% incurred by adults aged 65 years and older. This suggests that efforts to identify age-specific interventions to reduce hospitalisations are required. The per capita costs were greatest for individuals 85 years and older, 80–84 years old and under 5 years of age. In the context of an aging population, a specific focus on older adults may also be prudent in order to minimise the impact of AGI within this growing age group.

There was also a jump in total costs from ages 15–19 (3.8%) to ages 20–24 (6.7%), accounting for a 77% increase in total cost (Fig 1). This increase can be attributed to both population size and rate of gastroenteritis. There is a 56% increase in the population-weighted cost between the two age groups, with 15–19 year olds accounting for 6.2% of the population and 20–24 year olds accounting for 7.0%. Further, the per capita rate of gastroenteritis increases from 0.42 for 15–19 year olds to 1.3 for 20–24 years olds and remains at about 1 until age 35. It seems likely that this elevated rate of gastroenteritis, which occurs across the childbearing years, is related to contact with young children.

Where possible, the current study made use of nationally-representative datasets in order to improve the estimates of service utilisation and costs. In the 1999 study, an epidemiological study of only 2,811 individuals was used to estimate rates of GP consultations, ED visits and hospital admissions. In the current analysis, we estimated GP consultations from the Australia-wide BEACH survey data and ED visits and hospital admissions from national reports (population-level data). The cost of ED visits was originally based on the average cost of attending the Royal Children’s Hospital in Melbourne, while the current analysis used published ED costs representative of all public hospitals in Australia. We also applied a more conservative approach to the costs associated with medications (excluding over the counter medications and using generic brand costs).

Our study reported only direct healthcare costs in an effort to provide a conservative, but accurate, estimate of the financial burden associated with AGI. The Australian healthcare system is predominantly government-funded with the majority of costs borne by the public system; therefore health policy makers are interested in the financial burden on the public healthcare system. However, direct public healthcare costs represent only a small fraction of the total societal burden. Most COI studies have reported that direct costs account for about 20% of the total cost of AGI [12, 17, 20, 46] while one Canadian study reported it at 46.5% [18]. This highlights that our estimate of healthcare costs significantly underestimates the societal burden of AGI, which is consistent with a recent study that estimated substantial productivity losses due to AGI, including 13.1 million days of missed paid work each year in Australia [21]. If we assume that our estimate of direct costs accounts for 20% of the total burden, then estimated costs from AGI would AUD$1.8 billion. In addition to productivity losses, there are other societal costs that would inflate this estimate. For example, there are potentially significant opportunity costs, including the costs to isolate a patient presenting with AGI in hospital (ward closures, halting patient transports, etc.).

This study has several limitations. First, we used the best available data sources, but these were drawn from multiple studies spanning a range of years (e.g. NGSII survey conducted in 2008/9, BEACH dataset collected between April 2011 and March 2016, costing data from 2016), rather than a single study across a specific time period. While numbers were adjusted using relevant population data and the Health Price Index (HPI), it is possible this lead to some inaccuracy in the estimated costs. Further, the NGSII data, which was used to estimate the incidence of AGI, may have over-estimated the number of cases in children as data were collected in 2008–2009 and may not reflect the current community coverage of the rotavirus vaccine. Second, our estimate of costs for hospital admissions uses average costings based on AR-DRGs, representing costs associated with patients experiencing similar clinical conditions requiring similar hospital services. The most appropriate cost grouping is described as “Oesophagitis and gastroenteritis with/without catastrophic or severe complication or comorbidity” and is therefore not specific to AGI cases only. As a result, this cost estimate may over- or under-estimate the true cost of AGI hospitalisations. Similarly, the URG codes used to estimate ED costs are not AGI-specific. This analysis also did not account for ‘coning’ of pathology tests, which restricts payment for pathology tests to the three most expensive items when more than three tests are requested by a GP, as this was assumed to occur in only a small proportion of GP consultations; however, it may have resulted in a slight over-estimation of pathology costs. Third, the costs of some public hospital care in Australia are reimbursed by private health insurance if patients have relevant policies. The latest data from 2010–11 [47] indicated that 10.5% of patients in public hospitals were private patients (i.e. paid for by private health insurance). Hence the present study might have over-estimated publicly funded hospital care, but this would have been by 10–11% at most. Fourth, the data used to exclude private healthcare service utilisation (ED visits, hospital admissions and specialist visits) were based on data not specific to AGI and therefore, if the relationship between public and private service utilisation is different for AGI cases, it may have affected cost estimates. Finally, while we accounted for parameter uncertainty using sensitivity analyses, we did not report interval estimates as were unable to obtain confidence intervals for the majority of the input values used.

## Conclusion

While chronic diseases comprise a large cost burden on the healthcare system [48, 49], acute illnesses, including AGI, also impose substantial direct healthcare system costs.

The COI estimate from this study provides valuable information that can be used to assess the cost effectiveness of a range of interventions to prevent AGI, with a particular focus on young children and older adults. New targeted interventions to reduce hospitalisation rates among these high-risk groups could help reduce the financial burden.

## Supporting information

S1 Table Estimated GP consultations for AGI in 2016. (DOCX)

S2 Table Costs of prescription medications. (DOCX)

S3 Table Input variables for probabilistic sensitivity analyses including base case, minimum, and maximum values. (DOCX)

## References

[1] Tam CC, Rodrigues LC, Viviani L, Dodds JP, Evans MR, Hunter PR, et al Longitudinal study of infectious intestinal disease in the UK (IID2 study): incidence in the community and presenting to general practice. Gut. 2012 1;61(1):69–77. doi: 10.1136/gut.2011.238386 . Pubmed Central PMCID: 3230829. 21708822 10.1136/gut.2011.238386 PMC3230829

[2] Muller L, Korsgaard H, Ethelberg S. Burden of acute gastrointestinal illness in Denmark 2009: a population-based telephone survey. Epidemiology and Infection. 2011;140(02):290–8. 21470439 10.1017/S0950268811000471

[3] Herikstad H, Yang S, Van Gilder TJ, Vugia D, Hadler J, Blake P, et al A population-based estimate of the burden of diarrhoeal illness in the United States: FoodNet, 1996–7. Epidemiology and Infection. 2002;129(01). 10.1017/s0950268801006628 PMC2869879 12211601

[4] Majowicz SE, Hall G, Scallan E, Adak GK, Gauci C, Jones TF, et al A common, symptom-based case definition for gastroenteritis. Epidemiology and Infection. 2008;136(7):886–94. doi: 10.1017/S0950268807009375 17686196 10.1017/S0950268807009375 PMC2870876

[5] Hall G, McDonald L, Majowicz SE, Scallan E, Kirk M, Sockett P, et al Respiratory symptoms and the case definition of gastroenteritis: An international analysis of the potential impact on burden estimates. Epidemiology and Infection. 2010;138(1):117–24. doi: 10.1017/S0950268809990112 19493373 10.1017/S0950268809990112

[6] Ho SC, Chau PH, Fung PK, Sham A, Nelson EA, Sung J. Acute gastroenteritis in Hong Kong: a population-based telephone survey. Epidemiol Infect. 2010 7;138(7):982–91. doi: 10.1017/S0950268809991087 . 19925690 10.1017/S0950268809991087

[7] Scavia G, Baldinelli F, Busani L, Caprioli A. The burden of self-reported acute gastrointestinal illness in Italy: a retrospective survey, 2008–2009. Epidemiology and Infection. 2012 7;140(7):1193–206. doi: 10.1017/S0950268811002020 . Pubmed Central PMCID: 3365479. 22014077 10.1017/S0950268811002020 PMC3365479

[8] Baumann-Popczyk A, Sadkowska-Todys M, Rogalska J, Stefanoff P. Incidence of self-reported acute gastrointestinal infections in the community in Poland: a population-based study. Epidemiol Infect. 2012 7;140(7):1173–84. doi: 10.1017/S0950268811001853 . 21923971 10.1017/S0950268811001853

[9] Gibney K, Sinclair M, O’Toole J, Leder K. Establishing Australian Health-Based Targets for Microbial Water Quality—Research Report Adelaide, Australia: Water Quality Research Australia Ltd 2012 Contract No.: WQRA Project Number: 1004/09; NHMRC Grant Number: 546283.

[10] Kirk M, Glass K, Ford L, Brown K, Hall G. Foodborne illness in Australia—annual incidence circa 2010 Canberra: National Centre for Epidemiology and Population Health, Australian National University, 2014.

[11] Gibney K, O'Toole J, Sinclair M, Leder K. Disease burden of selected gastrointestinal pathogens in Australia, 2010. International Journal of Infectious Diseases; IJID. 2014;28:176–85. doi: 10.1016/j.ijid.2014.08.006 25281904 10.1016/j.ijid.2014.08.006

[12] Hellard ME, Sinclair MI, Harris AH, Kirk M, Fairley CK. Cost of community gastroenteritis. Journal of Gastroenterology and Hepatology (Australia). 2003;18(3):322–8. 10.1046/j.1440-1746.2003.02959.x 12603534

[13] Hall GV, Kirk MD, Ashbolt R, Stafford R, Lalor K, Bell R, et al Frequency of infectious gastrointestinal illness in Australia, 2002: Regional, seasonal and demographic variation. Epidemiology and Infection. 2006;134(1):111–8. doi: 10.1017/S0950268805004656 16409657 10.1017/S0950268805004656 PMC2870359

[14] Najnin N, Sinclair M, Forbes A, Leder K. Community based study to compare the incidence and health services utilization pyramid for gastrointestinal, respiratory and dermal symptoms. BMC health services research. 2012 7 23;12:211 doi: 10.1186/1472-6963-12-211 . Pubmed Central PMCID: 3411466. 22824457 10.1186/1472-6963-12-211 PMC3411466

[15] Dey A, Wang H, Menzies R, Macartney K. Changes in hospitalisations for acute gastroenteritis in Australia after the national rotavirus vaccination program. The Medical journal of Australia. 2012 10 15;197(8):453–7. . 23072242 10.5694/mja12.10062

[16] Reyes JF, Wood JG, Beutels P, Macartney K, McIntyre P, Menzies R, et al Beyond expectations: Post-implementation data shows rotavirus vaccination is likely cost-saving in Australia. Vaccine. 2017 1 05;35(2):345–52. doi: 10.1016/j.vaccine.2016.11.056 . 27916411 10.1016/j.vaccine.2016.11.056

[17] van den Brandhof WE, de Wit GA, de Wit MAS, van Duynhoven YTHP. Costs of gastroenteritis in The Netherlands. Epidemiology and Infection. 2004;132(2):211–21. 15061495 10.1017/s0950268803001559 PMC2870096

[18] Henson SJ, Majowicz SE, Masakure O, Sockett PN, MacDougall L, Edge VL, et al Estimation of the costs of acute gastrointestinal illness in British Columbia, Canada. Int J Food Microbiol. 2008 9 30;127(1–2):43–52. doi: 10.1016/j.ijfoodmicro.2008.06.007 . 18649966 10.1016/j.ijfoodmicro.2008.06.007

[19] Henson SJ, Majowicz SE, Masakure O, Sockett PN, MacDougall L, Edge VL, et al Corrigendum to “Estimation of the costs of acute gastrointestinal illness in British Columbia, Canada” [Int. J. Food Microbiol. 127 (1–2) (2008) 43–52]. International Journal of Food Microbiology. 2011;147(1):86. 10.1016/j.ijfoodmicro.2008.06.007 18649966

[20] Edelstein M, Merk H, Deogan C, Carnahan A, Wallensten A. Quantifying the incidence and cost of acute gastrointestinal illness in Sweden, 2013–2014. Epidemiol Infect. 2016 10;144(13):2831–9. doi: 10.1017/S0950268816000467 . 26964750 10.1017/S0950268816000467 PMC9150422

[21] Chen Y, Ford L, Hall G, Dobbins T, Kirk M. Healthcare utilization and lost productivity due to infectious gastroenteritis, results from a national cross-sectional survey Australia 2008–2009. Epidemiology and Infection. 2016;144(2):241–6. doi: 10.1017/S0950268815001375 26095130 10.1017/S0950268815001375

[22] XE. Current and Historical Rate Tables—2016-12-31 2016 [10 July 2017]. Available from: http://www.xe.com/currencytables/?from=AUD&date=2016-12-31.

[23] Hoang VM, Tran TA, Ha AD, Nguyen VH. Cost of hospitalization for foodborne diarrhea: A case study from Vietnam. Journal of Korean Medical Science. 2015;30:S178–S82. doi: 10.3346/jkms.2015.30.S2.S178 26617452 10.3346/jkms.2015.30.S2.S178 PMC4659871

[24] Hendrix N, Bar-Zeev N, Atherly D, Chikafa J, Mvula H, Wachepa R, et al The economic impact of childhood acute gastroenteritis on Malawian families and the healthcare system. BMJ Open. 2017;7(9). 10.1136/bmjopen-2017-017347 PMC5589001 28871025

[25] Fischer TK, Nielsen NM, Wohlfahrt J, Pærregaard A. Incidence and cost of rotavirus hospitalizations in Denmark. Emerging Infectious Diseases. 2007;13(6):855–9. doi: 10.3201/eid1306.061432 17553223 10.3201/eid1306.061432 PMC2792867

[26] BEACH. BEACH: Bettering the Evaluation and Care of Health. Gastroenteritis in general practice April 2006—March 2011 Sydney: The University of Sydney School of Public Health, Australian General Practice Statistics and Classification Centre, Family Medicine Research Centre, 2011.

[27] AIHW. Emergency department care 2014–15: Australian hospital statistics Canberra, Australia: Australian Institute of Health and Welfare, 2015 Contract No.: Cat. no. HSE 168.

[28] AIHW National Hospital Morbidity Database—Separation statistics by principal diagnosis (ICD−10−AM 8th edition), Australia, 2013−14 to 2014−15 [Internet]. Australian Institute of Health and Wellbeing. 2015. Available from: http://www.aihw.gov.au/hospitals-data/principal-diagnosis-data-cubes/#noteshttps://reporting.aihw.gov.au/Reports/openRVUrl.do?rsRID=SBIP%3A%2F%2FMETASERVER%2FAIHW%2FReleasedPublic%2FHospitals%2FReports%2FHDU_PDX%201315%20suppressed.srx%28Report%29.

[29] DH. Medicare Benefits Schedule Book Operating from 01 December 2016 Australian Government Department of Health, 2014.

[30] Pharmaceutical Benefits Scheme (PBS) [Internet]. Department of Health. 2017 [cited 16 Feb 2017 and 20 Feb 2017]. Available from: http://www.pbs.gov.au.

[31] IHPA. National Hospital Cost Data Collection, Public Hospitals Cost Report, Round 19 (Financial year 2014–15). Independent Hospital Pricing Authority, 2017.

[32] Peery AF, Dellon ES, Lund J, Crockett SD, McGowan CE, Bulsiewicz WJ, et al Burden of gastrointestinal disease in the United States: 2012 update. Gastroenterology. 2012 11;143(5):1179–87 e1-3. doi: 10.1053/j.gastro.2012.08.002 . Pubmed Central PMCID: PMC3480553. Epub 2012/08/14. eng. 22885331 10.1053/j.gastro.2012.08.002 PMC3480553

[33] ABS. 3101.0 Australian demographic statistics, June 2016 Canberra: Australian Bureau of Statistics, 2016.

[34] Quarterly Medicare Statistics: Table 2—National Figures [Internet]. Department of Health. 2017 [cited 20 April 2017]. Available from: http://health.gov.au/internet/main/publishing.nsf/Content/Quarterly-Medicare-Statistics.

[35] AIHW. Medical workforce 2010. Canberra: 2012 Contract No.: Cat no. HWL 47.

[36] AIHW National Hospital Morbidity Database—Separation statistics by AR−DRG (version 7.0), Australia, 2013−14 [Internet]. Australian Institute of Health and Wellbeing. 2014. Available from: https://reporting.aihw.gov.au/Reports/openRVUrl.do?rsRID=SBIP%3A%2F%2FMETASERVER%2FAIHW%2FReleasedPublic%2FHospitals%2FReports%2FHDU_AR-DRG%201314.srx%28Report%29.

[37] IHPA. National Hospital Cost Data Collection Australian Public Hospitals Cost Report 2013–2014 Round 18. Independent Hospital Pricing Authority, 2014.

[38] AIHW. Emergency department care 2013–14: Australian hospital statistics Canberra, Australia: Australian Institute of Health and Welfare, 2014 Contract No.: Cat. no. HSE 153.

[39] ABS. 4390.0—Private Hospitals, Australia, 2013–14 Canberra, Australia: Australian Bureau of Statistics, 2015.

[40] ABS. 4390.0—Private Hospitals, Australia, 2014–15 Canberra, Australia: Australian Bureau of Statistics, 2016.

[41] ABS. 4390.0—Private Hospitals, Australia, 2009–10 Canberra, Australia: Australian Bureau of Statistics, 2010.

[42] AIHW. Emergency department care 2013–14: Australian hospital statistics Canberra: Australian Institute of Health and Welfare, 2014.

[43] AIHW. Metadata Online Registry Canberra: Australian Institute of Health and Welfare, 2017.

[44] National Hospital Morbidity Database—separation statistics by principal diagnosis in ICD [Internet]. Australian Institute of Health and Wellbeing. 2017. Available from: http://www.aihw.gov.au/hospitals-data/principal-diagnosis-data-cubes/.

[45] AIHW. Health expenditure Australia 2014–15 Canberra, Australia: Australian Institute of Health and Welfare, 2016 Contract No.: Cat. no. HWE 67.

[46] Tam CC, O'Brien SJ. Economic Cost of Campylobacter, Norovirus and Rotavirus Disease in the United Kingdom. PLoS ONE. 2016 in press;11(2):e0138526 doi: 10.1371/journal.pone.0138526 26828435 10.1371/journal.pone.0138526 PMC4735491

[47] King D. Private patients in public hospitals Australian Health Service Alliance, Australian Centre for Health Research, 2013.

[48] Wilcox S. Chronic diseases in Australia: the case for changing course Background and policy paper. Melbourne: Australian Health Policy Collaboration, 2014.

[49] AIHW. 4.2 Chronic disease—Australia's biggest health challenge Canberra: Australian Institute of Health and Welfare, 2014.

