# Supplementary material for: Cost of gastroenteritis in Australia: A healthcare perspective
Source: PLoS One. 2018 Apr 12;13(4):e0195759. doi: 10.1371/journal.pone.0195759 (PMC5896984; doi:10.1371/journal.pone.0195759)
Supplement: S1 Table — (DOCX) [file pone.0195759.s001.docx]

S1 Table. Estimated GP consultations for AGI in 2016.

| Age group (years) | All GP consultations - % in age group [[1](#_ENREF_1)] | GP consultations (2016) ^a^ | AGI-related GP consultations per 100 GP consultations [[1](#_ENREF_1)] | AGI-related GP (2016) ^b^ |
| --- | --- | --- | --- | --- |
| 0-4 | 6.29% | 9,218,132 | 2.76 | 253966 |
| 5−9 | 2.78% | 4,065,870 | 2.61 | 105945 |
| 10−14 | 2.36% | 3,451,994 | 1.94 | 66897 |
| 15−19 | 3.50% | 5,123,202 | 1.86 | 95048 |
| 20−24 | 4.58% | 6,704,811 | 2.75 | 184647 |
| 25−29 | 5.21% | 7,630,469 | 2.39 | 182226 |
| 30−34 | 5.57% | 8,161,708 | 1.97 | 160431 |
| 35−39 | 5.49% | 8,041,838 | 1.68 | 135307 |
| 40−44 | 5.96% | 8,728,968 | 1.39 | 121685 |
| 45−49 | 6.11% | 8,957,809 | 1.11 | 99286 |
| 50−54 | 6.87% | 10,069,325 | 1.00 | 100194 |
| 55−59 | 6.96% | 10,192,827 | 0.90 | 91718 |
| 60−64 | 7.33% | 10,744,649 | 0.81 | 87480 |
| 65−69 | 7.57% | 11,093,965 | 0.45 | 50248 |
| 70−74 | 6.66% | 9,754,214 | 0.52 | 50854 |
| 75−79 | 6.05% | 8,859,432 | 0.42 | 36929 |
| 80−84 | 5.18% | 7,592,328 | 0.46 | 35113 |
| 85+ | 6.29% | 8,101,470 | 0.44 | 36021 |
| Total |  | 146,493,011 |  | 1,893,996 |

^a^ Number of GP consultations in each age group were estimated using the percentage in each age group (2011-2016) multiplied by the total number of GP consultations in 2016 (146,493,011; [[2](#_ENREF_2)]).

^b^ Calculated.

# References

1. BEACH. BEACH: Bettering the Evaluation and Care of Health. BEACH Survey Report - Gastroenteritis in general practice April 2011 - March 2016. Sydney: The University of Sydney School of Public Health, Australian General Practice Statistics and Classification Centre, Family Medicine Research Centre, 2016.

2. Quarterly Medicare Statistics: Table 2 - National Figures [Internet]. Department of Health. 2017 [cited 20 April 2017]. Available from: <http://health.gov.au/internet/main/publishing.nsf/Content/Quarterly-Medicare-Statistics>.
