# Supplementary material for: Cost of gastroenteritis in Australia: A healthcare perspective
Source: PLoS One. 2018 Apr 12;13(4):e0195759. doi: 10.1371/journal.pone.0195759 (PMC5896984; doi:10.1371/journal.pone.0195759)
Supplement: S2 Table — (DOCX) [file pone.0195759.s002.docx]

*S2 Table. Costs of prescription medications.*

| **Prescribed medication^a^** | **Prescriptions** | | **DPMQ^b^ (AUD)** | **Weighted ($)** | **Average cost (AUD)** | **Weighted cost (AUD)** |
| --- | --- | --- | --- | --- | --- | --- |
|  | **#** | **%** |  |  |  |  |
| **Maxolon** | 486 | 23.8% |  |  | $13.03 | $3.10 |
| NOS | 300 |  | n/a | $0.00 |  |  |
| Tablets 10mg | 153 |  | $12.22 | $10.05 |  |  |
| Injection 10mg 2ml | 33 |  | $16.80 | $2.98 |  |  |
| **Lomotil** | 220 | 10.8% |  |  | $12.87 | $1.38 |
| NOS | 4 |  | n/a | $0.00 |  |  |
| Tablets 2.5mg | 216 |  | $12.87 | $12.87 |  |  |
| **Flagyl** | 188 | 9.2% |  |  | $15.60 | $1.43 |
| NOS | 19 |  | n/a | $0.00 |  |  |
| Tablets 200mg | 18 |  | $12.36 | $1.32 |  |  |
| Suspension 200mg 5ml | 37 |  | $21.87 | $4.79 |  |  |
| Tablets 400mg | 114 |  | $14.07 | $9.49 |  |  |
| **Nexium hp7** | 186 | 9.1% | $42.02 | $42.02 | $42.02 | $3.82 |
| **Fasigyn** | 141 | 6.9% |  |  | $14.89 | $1.03 |
| NOS | 3 |  | n/a | $0.00 |  |  |
| Tablets 500mg | 138 |  | $14.89 | $14.89 |  |  |
| **Imodium** | 124 | 6.1% |  |  | $8.66 | $0.52 |
| NOS | 109 |  | n/a | $0.00 |  |  |
| Capsules 2mg | 7 |  | $18.55 | $8.66 |  |  |
| Melts tablets 2mg | 8 |  | not on PBS | $0.00 |  |  |
| **Stemetil** | 106 | 5.2% |  |  | $12.79 | $0.66 |
| Tablets 5mg | 96 |  | $12.18 | $11.03 |  |  |
| Injection 12.5mg 1ml | 9 |  | $20.77 | $1.76 |  |  |
| Suppositories 25mg | 1 |  | not on PBS | $0.00 |  |  |
| **Metronidazole systemic** | 86 | 4.2% |  |  | $15.77 | $0.66 |
| Oral/systemic NOS | 4 |  | n/a | $0.00 |  |  |
| Oral Tablets 200mg | 5 |  | $12.36 | $0.75 |  |  |
| Oral Tablets 400mg | 58 |  | $14.07 | $9.95 |  |  |
| Oral Liquid 200mg 5ml | 19 |  | $21.87 | $5.07 |  |  |
| **Buscopan** | 79 | 3.9% |  |  | $0.48 | $0.02 |
| NOS | 25 |  | n/a | $0.00 |  |  |
| Tablets 10mg | 39 |  | not on PBS | $0.00 |  |  |
| Injection 20mg 1ml | 1 |  | $25.79 | $0.48 |  |  |
| Tablets 20mg | 14 |  | not on PBS | $0.00 |  |  |
| **Ondansetron** | 59 | 2.9% |  |  | $26.94 | $0.78 |
| NOS | 28 |  | n/a | $0.00 |  |  |
| Tablets 4mg | 1 |  | $22.30 | $0.72 |  |  |
| Tablets 8mg | 3 |  | $28.67 | $2.77 |  |  |
| Wafers 4mg | 17 |  | $25.30 | $13.87 |  |  |
| Wafers 8mg | 9 |  | $31.67 | $9.19 |  |  |
| Injection 8mg 4ml | 1 |  | $11.70 | $0.38 |  |  |
| **Gastro-stop** | 56 | 2.7% |  |  | $18.55 | $0.51 |
| Capsules 2mg | 56 |  | $18.55 | $18.55 |  |  |
| **Tinidazole** | 49 | 2.4% |  |  | $14.89 | $0.36 |
| NOS | 1 |  | n/a | $0.00 |  |  |
| Tablets 500mg | 48 |  | $14.89 | $14.89 |  |  |
| **Nexium** | 48 | 2.3% |  |  | $25.74 | $0.60 |
| NOS | 4 |  | n/a | $0.00 |  |  |
| Tablets 20mg | 19 |  | $22.07 | $9.53 |  |  |
| IV injection 42.5mg 5ml | 1 |  | not on PBS | $0.00 |  |  |
| Tablets 40mg | 24 |  | $29.71 | $16.21 |  |  |
| **Metoclopramide** | 45 | 2.2% |  |  | $12.55 | $0.28 |
| NOS | 31 |  | n/a | $0.00 |  |  |
| Tablets 10mg | 13 |  | $12.22 | $11.35 |  |  |
| Injection 10mg 2ml | 1 |  | $16.80 | $1.20 |  |  |
| **Zofran** | 44 | 2.2% |  |  | $26.00 | $0.56 |
| NOS | 21 |  | n/a | $0.00 |  |  |
| Tablets 4mg | 1 |  | $22.30 | $0.97 |  |  |
| Wafers 4mg | 19 |  | $25.30 | $20.90 |  |  |
| Wafers 8mg | 3 |  | $31.67 | $4.13 |  |  |
| **Norfloxacin** | 27 | 1.3% |  |  | $14.67 | $0.19 |
| Tablets 400mg | 27 |  | $14.67 | $14.67 |  |  |
| **Panadeine forte** | 27 | 1.3% |  |  | $12.44 | $0.16 |
| Tablets 530mg | 27 |  | $12.44 | $12.44 |  |  |
| **Repalyte sachets** | 27 | 1.3% | $15.90 | $15.90 | $15.90 | $0.21 |
| **Pramin** | 25 | 1.2% |  |  | $12.22 | $0.15 |
| Tablets 10mg | 25 |  | $12.22 | $12.22 |  |  |
| **Noroxin** | 22 | 1.1% |  |  | $14.67 | $0.16 |
| Tablets 400mg | 22 |  | $14.67 | $14.67 |  |  |
| **TOTAL** | 2045 | 100% |  |  |  | $16.59 |

^a^Top 20 prescribed medications were determined from Table 7.2.5.2 and individual drug products were determined from Table 7.2.4 [[1](#_ENREF_1)]. NOS=not otherwise specified.

**^b^**Costs (DPMQ; Dispensed Price for Maximum Quantity) were obtained from the Pharmaceutical Benefits Scheme website [[2](#_ENREF_2)].

# References

1. BEACH. BEACH: Bettering the Evaluation and Care of Health. BEACH Survey Report - Gastroenteritis in general practice April 2011 - March 2016. Sydney: The University of Sydney School of Public Health, Australian General Practice Statistics and Classification Centre, Family Medicine Research Centre, 2016.

2. Pharmaceutical Benefits Scheme (PBS) [Internet]. Department of Health. 2017 [cited 30 August 2017]. Available from: <http://www.pbs.gov.au>.
