# Supplementary material for: Cost of gastroenteritis in Australia: A healthcare perspective
Source: PLoS One. 2018 Apr 12;13(4):e0195759. doi: 10.1371/journal.pone.0195759 (PMC5896984; doi:10.1371/journal.pone.0195759)
Supplement: S3 Table — (DOCX) [file pone.0195759.s003.docx]

*S3 Table. Input variables for probabilistic sensitivity analyses including base case, minimum, and maximum values^a^.*

| **Variable** | **Base case** | **Minimum** | **Maximum** |
| --- | --- | --- | --- |
| GP visits | 1,893,996 | 1,420,497 | 2,367,495 |
| GP visits (minimum and maximum = 95% CI) | 1,893,996 | 1,692,012 | 2,095,979 |
| Cost of GP visit | $26.50 | $19.87 | $33.12 |
| GP visits with prescription medication | 793,984 | 595,488 | 992,480 |
| Cost of prescription medication | $16.59 | $12.44 | $20.74 |
| GP visits with referral to specialist | 10,775 | 8,081 | 13,469 |
| Cost of specialist visit | $118.23 | $88.67 | $147.78 |
| GP visits with referral for pathology test | 605,127 | 453,845 | 756,409 |
| Cost of pathology test | $40.80 | $30.60 | $51.01 |
| GP visits with referral for imaging | 9,337 | 7,003 | 11,672 |
| Cost of imaging | $128.89 | $96.66 | $161.11 |
| Hospital admissions | 60,125 | 45,094 | 75,156 |
| Cost of hospital admission | $3,407.55 | $2,555.66 | $4,259.44 |
| ED visits | 120,255 | 90,191 | 150,319 |
| Cost of ED visit | $527.58 | $395.68 | $659.47 |

^a^ Unless otherwise indicated, minimum and maximum values calculated as ±25% of base case value.
